# Congenital arteriovenous malformation (ISSVA classification for vascular anomalies)

**DOI:** 10.11604/pamj.2023.46.103.41202

**Published:** 2023-12-12

**Authors:** Subrat Narendra Samal, Snehal Subrat Samal

**Affiliations:** 1 Department of Musculoskeletal Physiotherapy, Ravi Nair Physiotherapy College, Datta Meghe Institute of Higher Education and Research, Sawangi, Meghe, Wardha, Maharashtra, India,; 2 Department of Neuro Physiotherapy, Ravi Nair Physiotherapy College, Datta Meghe Institute of Higher Education and Research, Sawangi, Meghe, Wardha, Maharashtra, India

**Keywords:** Haemangioma, malformation, vascular anomalies

## Image in medicine

Presenting a Magnetic Resonance Imaging (MRI) finding of a 7 year, 5-month-old male who was brought to our rural hospital by his father with chief complaints of swelling over his right hand since birth. His father narrated that the swelling is increasing significantly and the child has tenderness over swelling. The child underwent an angioembolism 1 month ago and has been brought for re-embolism. Now sclerotherapy has been planned for the patient. The physiotherapist received a call for a decreased range of motion at the elbow joint. Another haemangioma is also found in the forearm. MRI findings (sagittal T1W, T2W, STIR/PD) noted an altered signal intensity oval lesion of size 5.4 x 5.6 x 6.2 cm appearing in the anterior intermuscular compartment and deep subcutaneous plane of the right arm displacing underlying muscles. Multiple prominent vascular channels are noted in the subcutaneous plane of the right arm. A similar signal with intensity was also noted in the intermuscular compartment of the proximal, a third of the right forearm. The prevalence of Arteriovenous Malformations (AVM) is 1 per 100,000 populations per year. The patient is undergoing medications and physiotherapy rehabilitation exercises. The physiotherapy protocol started early and is continuing. Early medication and rehabilitation are the way to recover the range of motion in such patients.

**Figure 1:**
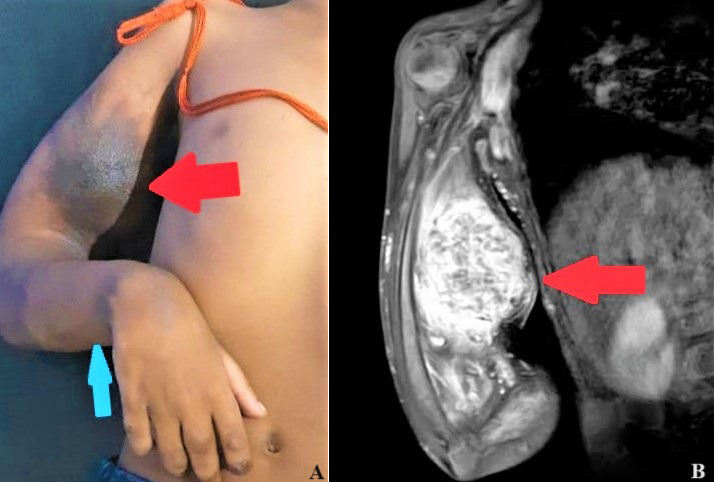
A) Magnetic Resonance Imaging (MRI) findings of bulging and blue-blackish discolouration, red arrows show oval lesion and blue arrow show skin blue colouration at intermuscular compartment of proximal 1/3^rd^ of right forearm; B) MRI findings of arm arteriovenous malformation- red arrows show oval lesion of arteriovenous malformation

